# Municipalities’ organisational capacity to support the implementation of the Multi-Sector Nutrition Plan in Burkina Faso

**DOI:** 10.1080/16549716.2021.1979279

**Published:** 2021-09-29

**Authors:** Dieudonne Diasso, Maimouna Halidou Doudou, Mohamed Cheikh Levrak, Holly Dente Sedutto, Aly Savadogo

**Affiliations:** a Department of Biochemistry and Microbiology, University Joseph Ki-Zerbo, Ouagadougou, Burkina-Faso; b Department of Public Health, African Private University for Development (UPAD), Niamey, Niger; c United Nations Entity for Nutrition (UN Nutrition), Renewed Efforts Against Child Hunger, World Food Programme (WFP), United Nations Renewed Efforts Against Child Hunger, Regional Bureau Dakar, Dakar, Senegal; d United Nations Entity for Nutrition (UN Nutrition), Renewed Efforts against Child Hunger Special Project, Rome, Italy

**Keywords:** Rural municipalities, multi-sector nutrition plan, capacity dimension, needs assessment, Burkina Faso

## Abstract

The Government of Burkina Faso committed to the multi-sector approach on nutrition in 2014 and has conducted the development of a Multi-Sector Nutrition Plan 2020–2024. This study aims to understand and analyse the Nutrition organizational capacities at the municipal level to support the scaling up of interventions within the National Multi-Sector Nutrition Plan. A qualitative study was conducted at the end of 2017, based on the framework for nutrition capacity developed by the United Nations Network Secretariat in collaboration with five funding agencies, to assess the organizational capacity dimension. Data collection consisted of focus groups and information collection through workshops with key informants. In total, 22 rural municipalities were targeted and 152 key informants were involved, including mayors, municipal councillors, members of the village development committee, and local technical agents in charge of agriculture, livestock and health. The gaps identified were poor integration of nutrition into local development strategic plans, less evolved coordination on nutrition, weak development of nutrition community approaches and dependence on the state budget matched to a non-existent budget monitoring system. The findings showed an unequal distribution and limited number of technical agents to cover villages within a given municipality, inadequate skills to support services expansions such as water and sanitation, health, agriculture and livestock. In addition, no reference was made to monitoring and evaluation, accountability or sharing information. The main capacity needs on nutrition are the transfer of technical competencies from the regional to the municipal level, the strengthening of technical skills on nutrition, and the setting up of an integrated data collection system involving key players. The identification of needs and opportunities and the newly finalized guide on nutrition integration into local development plans and strategies are useful to drive change for multisectoral implementation.

## Background

The highest proportion of stunted children live in low- and middle-income countries, located in the African continent, with 35.6% of children under 5 years stunted [1]. In Burkina Faso, 52% of the adult population suffered from stunting during childhood [2], and infant mortality due to undernutrition also reduced the rate of productive population by 13.5% [2]. The fight against malnutrition and the struggle for child survival requires the involvement of various sectors and multiple interventions. There are dietary diversification, protection and support for recommended infant and young child feeding practices, social protection, management of acute malnutrition, access to safe water and sanitation, adequate health services, better accommodation, prevention of early pregnancy and optimal birth spacing [3–5].

The Government of Burkina Faso embraced the United Nations REACH initiative (Renewed Efforts Against Child Hunger and undernutrition) in 2014, and took advantage of opportunities to improve coordination of the multi-sector approach to nutrition and other aspects of nutrition governance [6,7]. This initiative, together with other advocacy actions, enabled the development of a Multi-Sector Nutrition Policy 2020–2029. In March 2017, a workshop was held to support the future operationalization of the Multi-Sector Nutrition Plan with an onus on scaling up of nutrition interventions at the municipal level; this attracted key local actors.

Most capacity development of municipalities in Burkina Faso is often supported by various programs, such as territorial development, and development of local economies, in order to help them to ensure better social and political ownership of decentralization.

Decentralization is considered as the transfer of administrative power and resources to subnational governance structures for program management and evaluation [8], planning and implementation [9]. It is often predicated that decentralization improves the quality of public services [10]. The translation of political ambitions to concrete impacts hinges on various types of capacities, but no detailed knowledge of capacities at the municipality level are available.

In addition, the process of multisectoral operationalization seems less developed or not explicit; and what it implies, requires and how to implement it are still vague [11].

Weak nutrition capacity has been noted as a limiting factor in the implementation of a large-scale program for several decades [12]. To address these constraints, it is important to have a common understanding of the capacities needed, those that already exist, as well as those capacities that must be strengthened [12]. There was a consensus among stakeholders to conduct this assessment study at the municipality level following the national consultation workshop.

The objective of this study was to understand and analyse the nutrition organizational capacities at the municipal level, and to identify opportunities to scale up interventions in support of the operationalization of the National Multi-Sector Nutrition Plan.

## Methods

This municipal-level study is part of a larger capacity needs assessment that was conducted at the central and regional levels [13,14]. A series of tools and methods were used to analyze capacities, namely: (a) stakeholder mapping; (b) stakeholder analysis; (c) checklist for capacity areas; (d) focus groups interview; and (e) identification of capacity development needs based on the differences between current capacity and the desired future capacity [15].

In order to build collaborative and inclusive actions, the process was a participatory multisectoral and multidisciplinary Technical Evaluation Committee created by the Ministry of Health, and supported by the REACH. One of the strengths of this process was real-time feedback to the different stakeholders [16], and their commitment to use the results of the evaluation for decision-making purposes. The committee members were fully engaged at all steps in key decisions and orientation of the study, and they were trained on the tools and resources of the Framework for Nutrition Capacity.

### Study conceptual and analytical framework

The framework for nutrition capacity jointly developed by the United Nations Network Secretariat and five United Nations founding agencies (Food and Agriculture Organization, World Health Organization, World Food Programme, United Nations International Children’s Emergency Fund and International Fund for Agricultural Development) [17] was applied for this research study. The framework comprised two components, an orientation guide and a tool and resource kit. The framework highlights three dimensions of capacities: (a) the enabling environment; (b) organizational; and (c) individual. Four areas of capacities (policies, programs and frameworks; resources and infrastructure; coordination and partnerships; and evidence-based decision-making) can be applied within each dimension of capacity [17]. Furthermore, the tools and resources proposed can be adapted to different needs, to country contexts and assessment objectives [17]. In addition, this led to the development of a checklist for capacity areas that was used to establish some analytical criteria for the capacity assessment undertaken at the municipality level (Table 1). The analysis of organizational factors was the main dimension considered which guided the articulation of a list of capacity areas, which in turn, helped to identify some assessment topics focusing on five key themes.

**Table 1.** Analytical framework established for capacity assessment at municipal level

Capacity dimensions	Capacity Area	Assessment topic/criteria
Organizational	Policies, programmes and frameworks	Existence of plan or document and nutrition integration
	Resources and Infrastructures	Availability of adequate skills to support services expansion
		Partnership and resource mobilization
	Coordination and partnership	Local coordination bodies for nutrition sensitive and/or nutrition specific actions
	Evidence-based decision making	Routine data collection and monitoring and evaluation

### Municipalities and organization identification and selection

Two municipalities per region (13 regions in total) were selected in this qualitative research study. For this purpose, a random sampling was made with the only criterion being distanced from the principal town by at most 75 km, leading to the choice of 26 municipalities.

The stakeholder mapping made it possible to draw up a list of all the key organizations and structures (who does what, where and how?) [16], thus obtaining a first list of the potential key organizations and structures to participate in the study. In addition, the stakeholder analysis tool [16] made it possible to retain organizations and structures combining strong resources/capacities to facilitate the achievement of shared results and the interests pursued for the Multi-Sector Nutrition Plan.

### Data collection

A focus group guide with questions and sub-questions on the main topics of the analytical framework in Table 1, was used to collect information: (a) Existence of plan or document and nutrition integration; (b) local coordination bodies for nutrition sensitive and/or nutrition-specific actions; (c) partnership and resource mobilization; (d) availability of adequate skills to support services expansion; and (e) routine data collection and monitoring and evaluation. Data collection consisted of the organization of focus groups and information collection through workshops with key informants.

Data collection in the field took place from 19 November 2017 to 5 December 2017, and was carried out by trained agents from ministerial departments (water and sanitation, agriculture, health, education and social protection). Three teams, each composed of three people, were deployed to the 13 regions, with an average distribution of 4 regions per team to cover the 26 municipalities (2 per region). Discussion groups with six to eight key informants, depending on the municipality were organized. Careful note-taking was completed by two people, while the third person with strong experience led the focus group. Follow-ups with the municipalities interviewed were carried out when information was missing or needed to be clarified after the interviews. Note-taking was preferred for audio recording as most of the municipalities that participated expressed a clear preference for the interviews to be conducted without a tape recorder. Following the focus group, the preliminary results were presented and vetted at two validation workshops: (a) a technical pre-validation workshop with the participation of 10 people, and (b) a national consultation that brought together 41 stakeholders. These workshops made it possible to provide clarification and obtain additional contributions in order to refine the results.

### Data analysis

A content analysis of the collected data after the focus group was done based on the keywords and ideas, and coding was carried out. A manual processing was employed using a deductive approach. It involves the process of carefully reading and rereading the collected qualitative data [18]. An analysis grid on the current situation (strengths and weaknesses) previously designed was competed for each municipality. Discussions were conducted within each team and between the three teams by reaching a consensus on the content of each municipal grid. From each grid validated, a synthesis of the common points was done. Subsequently, the strengths and weaknesses of the existing situation were listed and analyzed for each of the five criteria evaluated. Thereby, differences between the existing situation and the desired or future situation made it possible to identify the gaps for capacity development, and to identify the needs highlighted as well as the existing opportunities.

## Results

### Presentation of municipalities and participants

The thirteen regions in Burkina Faso are subdivided into 45 provinces, under the leadership of high commissioners. Furthermore, the country has 370 departments, which are governed by prefects under the supervision of the provinces. The departments are divided into 49 urban communes or municipalities and 302 rural communes under the direction of the mayors. For this study, 22 out of the 26 focus groups planned were organized in rural municipalities, including two municipalities by region (Table 2). The mapping and stakeholder analysis enabled the participation of a total of 152 key informants in the focus group for all the municipalities covered. The key informants were mayors, municipal councillors, members of the village development committees and the agents of decentralized technical services such as agriculture extension workers, livestock technicians and health workers.

**Table 2.** Targeted regions and municipalities for data collection

Region	Municipalities	Number of focus group discussions
Nord	Tangaye, Oula	2
Sahel	Bani, Seytenga	2
Est	Diabo	1
Centre ouest	Zamo, Zawara	2
Centre est	Lalgaye, Bagré	2
Centre nord	Mané, Pissila	2
Centre sud	Guiba, Guiaro	2
Sud-ouest	Lorepéni, Perigban	2
Boucle du mouhoun	Bourasso, Douroula	2
Cascades	Sidéradougou	1
Plateau central	Loumbila, Zitenga	2
Hauts bassins	Bama, Toussiana	2
Total number of focus group discussions		22

### Assessment diagnostic results

The organizational dimension capacity results are summarized in Table 3, highlighting the strengths and weaknesses of the current situation experienced by the municipalities.

**Table 3.** Municipalities’ diagnostic results highlighting strengths and weaknesses

Key diagnostic criteria	22 municipalities current situation	
	*Strengh*	*Weakness*
Existence of plan or document and nutrition integration	Communal development plan drawn up and taking into account several sectors: food security, tourism, land, gender, sport and culture, transport, environment, health, and education. Existing support documents. Involvement of local technical agents for Communal Development Plan elaboration and implementation.	Plan’s financing needs are enormous compared to existing resources. Plan not yet aligned with the national economic and social development plan. No specific consideration for nutrition with a budget line.
Local nutrition coordination bodies	Municipal council sessions are held regularly. Some coordination bodies exist and are fairly organized: eg existence in municipalities, Coordination of School Management Committees with terms of reference defining roles and attributions.	Terms of reference for local structures not clearly defined and seem rather informal. Weak presence of an umbrella organization (e.g. agriculture, agrifood) and very informal. Lack of coordination body for nutrition.
Partnership and resource mobilization	Resource mobilization strategy/initiative: national/international inter-municipality twinning, advocacy, round table with municipality citizens, rentals on market infrastructure, taxes. Community sensitization on the town hall projects for fundraising. Strong involvement of municipal services for tax collection and strengthening of collaboration with technical services. Existence of cooperation development projects and programs. Availability of monitoring and audit capacities (accountant and procurement manager).	Difficulties to gather more resources because there are many priorities. Weak budget monitoring system. Insufficient number of technical and financial partners. Low municipalities’ own funds. Weak intertechnical service collaborations. Strong dependence on the state budget through the permanent fund.
Availability of adequate skills to support services expansion	Capacity building taken into account in the communal development plans. Existence of some structures, which support the municipality in capacity building. Existence of regional associations able to ensure skills enhancement.	Unequal distribution and insufficiency of decentralized technical staff at the local structures and villages level. Insufficient staff trained in nutrition (a few health workers and rarely among agriculture and livestock workers). Low existence of associations and community groups. Limited community-based nutrition and development approaches.
Routine data collection and monitoring and evaluation	Data on animal health (zoonosis). Disease screening, data on treatment of malnutrition, consultations, epidemiological surveillance, vaccination campaigns, etc. Data on animal foodstuff inspection, and on agricultural campaign. Agricultural production situation in the municipality. Data transmission to the prefecture, town hall and technical departments of the region; Feedback from the hierarchy.	No direct transmission, dissemination or reporting to the town hall. Lack of analysis at the operational and local level. Poor decision-making and action following data analysis. Health and education, routine and monitoring and evaluation data are collected and sent to the town hall. Data from other sectors are either transmitted to the prefecture or to the higher grade in the hierarchy (Provincial Directorate).

### Existence of plan or document and nutrition integration

All municipalities have Communal Development Plans (CDP) which take into account several aspects of development (agriculture, livestock, water, transport, environment, health, education, etc.). These plans were elaborated in 2014 or 2015, with expiry in 2018 or 2019, and needed to be reviewed. In general, sensitive and specific nutritional interventions were not sufficiently integrated into the CDPs. When integrated, they are primarily extended through the health sector targeting malnourished children and pregnant or lactating women with activities such as prenatal consultations, maternal nutrition counselling and provision of materials (e.g. weighing scales, measuring tape). The reasons given underlined by the key respondents for the limited integration of nutrition in the CDPs were mainly weak technical capacities or skills in nutrition (17/22), lack of financial resources (13/22), and poor local expertise in planning (5/22). Unanimously, the municipalities indicated that the CDP financing needs were enormous compared to the available resources.

### Local nutrition coordination bodies

The majority of the municipalities do not have a sensitive and specific nutrition coordination body; however, specific initiatives were identified in certain geographic areas. For example, there is a water, sanitation and hygiene (WASH) focal point at the town hall in the municipality of Tangaye in the North, and Seytenga in the Sahel. This focal point coordinates the ‘water users associations,’ created at the village level. A member of the village development committee in the municipality of Zambo said:

*‘In our municipality, local associations exist; we have local associations for wild fruits, and also for fisheries’.*

### Partnership and resource mobilization

Various partners and development projects and programs were identified in the municipalities of Burkina Faso. These included decentralized cooperation, environmental protection and humanitarian assistance for children, including efforts to protect fundamental human rights such as the right to food, quality education, and so on. Nonetheless, the municipality-level actors all reported a lack of technical and financial partners, resources and weak collaboration between technical services for agriculture, health and education, among other areas. Municipality funds were considered low and there were many priorities, further compounding the situation. The common local priorities listed were new health centers, improving livestock market, ending of schools under straw huts, municipal infrastructure development, implementation of police station plan, civil security and enrichment of infant flour. Some municipalities have initiated innovative strategies to mobilize more resources. In this regard, two mayors explained that:

- *‘We have initiated taxes to be collected on raw materials, trucks, wood, venues [and] wandering animals’.*
- *‘The local security issues have negatively affected investments, projects and program implementation in the North and the Sahel region’.*

### Availability of adequate skills to support services expansion

Many municipalities expressed a lack of staff trained in nutrition. As such, exclaimed a head nurse:

In our municipality, most often, health agents are trained on child malnutrition and illnesses.

The assessment also drew into focus the role of communities in nutrition capacity development, as they are the ultimate beneficiaries of nutrition services. A member of the focus group said:

There are not enough community animators (or agents) here, and most of the associations in the community are not trained on nutrition issues, they are only trained on how to plant crops.

The existence of community agents (nutrition change agents) was very low within municipalities (18% (4 out of 22)), although some initiatives were observed:

- The ‘schools for husbands’ in the Pissila municipality of the Center-North region was a strategy to involve men in reproductive health issues.
- There were ‘Mothers leaders in Infant and Young Child Feeding (IYCF)’ and acute malnutrition community care, again in the Pissila municipality located in the Center-North, who educate and support each other by sharing a range of experiences related to community-level nutrition issues.
- IYCF Practice Monitoring and Learning Groups (GASPA) were found in the Diabo municipality in the East, Douroula in the Boucle de Mouhoun and Toussiana in the Hauts Bassins. These were groups of women who promoted good practices as part of efforts to implement the IYCF plan.

In addition, information collected from the Health Promotion and Education Directorate of the Ministry of Health indicated 17,628 community-based health workers (ASBC), were trained in October 2016 in the 13 regions (a total of 68/72 health districts covered) on the government budget in partnership with the Global Fund foundation. An agent from the health promotion directorate on the subject said:

Some ASBCs are no longer functional either on account of resignations, deaths or incompetence due to non-compliance with recruitment guidelines. There is subsequently a need for refresher training and training of new recruits.

Moreover, many Non-Governmental Organization and Associations led initiatives in short, medium and long term, to strengthen local capacities, such as nutrition and feeding knowledge and practices, hygiene and sanitation, local fortified foods production and marketing.

### Routine data collection and monitoring and evaluation

It was noted in the focus group discussions that the data were collected by the local technical structures, and transmitted to higher levels in the hierarchy (regional and central) often without triangulating the information. Furthermore, there was no systematic feedback on the implementation of CDP activities to the town hall. In this sense, one of the local elected officials emphasized:

Although the sectors have activities in the CDPs, they do not report to the town hall. Most often, it is the education and health sectors that share information with the town hall.

Moreover, an agricultural agent underscored:

Capacity at the community level is limited due to lack of supervision and adequate training in the fields of nutrition or food systems and nutrition for extension agents and agricultural advisory support/livestock advisory support. In addition to this, we are understaffed to cover all the villages and hamlets.

## Needs and opportunities identified

The differences between current capacity and the desired future capacity made it possible to identify priority needs such as the development of nutrition community approaches and strengthening the competence of technical agents in nutrition, and opportunities to support the multisectoral approach to nutrition (Table 4).

**Table 4.** Identified priority needs and opportunities for multi-sector nutrition actions

Summary of findings	Needs identified	Opportunities
Poor consideration of nutrition in municipal strategic documents	Increase nutrition integration into municipal development plans	Existence of nutrition integration guide to PCDs produced by REACH United Nations Burkina in partnership with the Ministry of Health
	Sufficient resources mobilization for the implementation of activities in priority sectors	Political commitment at the highest level of the state. The President is the Champion of the African Leaders for Nutrition of the african development bank
Local coordination bodies for nutrition sensitive and specific actions obsolete and underdeveloped	Consultation body for the local technical services by the municipality around various topic – sectoral and global consultation	Existence of PCDs. Existence of consultation body at the municipalities level that can integrate other topic and actors.
	Community associations/groups and new nutrition community approaches creation and development	Support to local capacity building initiatives by NGOs and United Nations agencies. Existence of mother-to-mother support groups with supervision from ASBCs
	Acceleration of umbrella organization establishment and the agricultural, agro-food sector	Existence of farmers group: farmer’s confederation, agricultural professional’s federation and the national federation of Agrifood and Processing Industries.
Partnership and resource mobilization in difficulty	Skills transfers increase from the regional to the municipal level	Good collaboration between health structures and municipalities within the current transfer of health centers management system; Existence of goldmine in several areas of the country which are potential sources through corporate social responsibility
	Networking and partnership skills improving	
Lack of adequate skills to support the services expansion	Strengthening the technical competence in nutrition of health, agriculture, livestock, environment agents and town halls services)	Recruitment of nutritionists by the Ministry of Health to make them available to regional health directorates in order to provide support at all levels within the regions.
Weak accountability and information sharing	Dissemination of good practices	Routine data collection support by United Nations Agencies in Burkina Faso, via ENDOS
	Development of integrated data collection system involving key players	
	Data sharing for guidance and decision making	

ASBCs = Community-based health workers; CDP = Communal development plan; ENDOS = Health data warehouse; NGOs = Non-governmental organizations; REACH = Renewed Efforts Against Child Hunger

## Discussion

The current study of the 22 municipalities used the new framework for assessing nutrition capacities [17], and made it possible to identify the gaps and needs for the organizational dimension. This, in turn, helped to identify the support required to implement Burkina Faso’s Multi-Sector Nutrition Plan [19,20].

The findings showed the involvement of local government technical agents during the development and implementation of the CDPs, but less consideration was afforded to nutrition compared to other sectors. There was also no specific budget line for Nutrition. The main reasons given were: weak human resource capacities for nutrition, a lack of financial resources, and limited local expertise in planning. A different qualitative study carried out in five other municipalities in Burkina Faso corroborated these results [21]. Several research conducted in nine sub-Saharan African countries [22], India and Tanzania communities [23], Ethiopia and Senegal [24], district or sub-national in Indonesia [25], and Vietnam provinces [26] explained that the weakness in nutrition integration was due to limited capacities for locally elected representatives on local planning and nutrition. In addition, the CDPs financing needs were described as enormous compared to the existing resources, hindering the successful implementation of the action plans. In Bolivia, limited funding, poorly trained staff and understaffing were highlighted as negative factors for municipal capacity to invest in human development [27]. Another potential explanation could be that nutrition lacks a distinct institutional home unlike other technical sectors (e.g. agriculture, health, education), which had a designated ministry to address related issues.

This research showed that although there was some local initiatives to coordinate water, fisheries and forestry issues, local or communal bodies for coordinating nutrition-specific and nutrition-sensitive actions were obsolete and underdeveloped. Moreover, a national study indicated weak collaboration between the regional technical directorates and the regional nutrition coordination bodies in the 13 regions of Burkina Faso [13].

The state of coordination at the municipality level reflected the regional-level inadequacies. Some authors mentioned that the absence of coordination mechanisms, marked by the fragmentation of the stakeholders were not favourable for dialogue or bolstering commitment [28–30]. Weak nutrition community approaches were underlined as another limiting factor. In addition, the weak presence and very low number of formalized organizations (e.g. agriculture, agrifood), which coordinate activities and/or pool resources, contribute to the situation. A study carried out in Vietnam provinces showed that the involvement of umbrella organizations was viewed as positively influencing outcomes and played an important role in nutrition [9].

However, we identified some good practices about endogenous financial resource mobilization strategies such as national/international inter-municipality twinning, round tables with municipality citizens, rentals on market infrastructure and taxes. Similar good practices such as property tax, meat inspection fee and health facility user charges were reported in Kongwa district in Tanzania with 14 wards increased the possibility of deciding on how to use them for the implementation of health services [31]. The study reported high dependence on the central government’s budget as well as insufficient municipality funds, intensified by many development priorities and a weak budget monitoring system. A similar case was observed in Cote d’Ivoire where local governments were faced with limited financial resources to deliver health services [32]. There is a push for countries engaged in the Scaling Up Nutrition (SUN) Movement to mobilize domestic financing [19], and decentralization confers autonomy in the mobilization of financial resources from local sources and the possibility of deciding on how to use them [22]. In addition, the weakness of inter-technical service collaboration was widely raised and can certainly be linked to the fact that the mechanisms for assigning clear roles and responsibilities remain insufficient [33]. A study conducted in six municipalities of Burkina Faso found a lack of formal coordination mechanism between health centres and local governments [34]. This phenomenon could be partially explained by a lack of leadership [9,22,35]. A review of health system decentralization in Ghana, Zambia, Uganda and the Philippines showed that the financing, service organizations, and governance were influenced by the type and level of decentralization [36].

Another major finding from our research was the unavailability of technical adequate skills to support the expansion of nutrition services as well as others, such as water and sanitation, health, agriculture and livestock that are crucial for safeguarding the nutrition of the Burkinabè people. The unequal distribution and low number of personnel for such technical services coupled with low coverage of villages were observed. Similar findings were reported from 10 municipalities in Bolivia [27] and Uganda [37], indicating a lack of capacity and personnel to exercise responsibility for service delivery on nutrition or health services. This situation can be explained by low recruitment and the fact that agents prefer to be posted at regional and provincial levels where they can earn higher wages and have brighter prospects for advancing their career. A study on local and intersectoral experiences in water, education and health, carried out in 12 African countries, indicated that the main obstacles for the effective transfer of skills to municipal bodies are the unsuccessful retention of financial and human resources at the central level and insufficient training of local actors [38].

Additionally, various collection of routine data in the key sectors, agriculture, animal, human health was exposed by the municipalities, but monitoring and evaluation, accountability and information sharing was reportedly weak. Non-transmission limited dissemination and reporting to the town hall was affirmed by most of the technical services; data were sent directly to provinces or regions. This arrangement directly contributes to the lack of data collected and analysed at the local level and creates information asymmetries between the province or regional level and the municipality level [39]. The situation could be due to the lack of a culture of accountability [34,40], and to the low local capacity to develop management mechanisms and involve key actors in the municipality [20,39]. Efforts to revisit communication and reporting lines and build managerial capacity could empower the decentralized approach, strengthening feedback loops between the central and local government authorities. Similarly, increased attention to country-level knowledge management systems, platforms and practices could help to systematically address these shortcomings and help to support peer learning and replicate good practices at the municipality level. This, in turn, helps to optimise the use of existing resources.

This study has various limitations. Considering the plethora of rural municipalities in the country, the focus groups covered a small number (22) of municipalities selected at random, but it allowed us to understand, for our research purposes, the capacities in certain municipalities. This qualitative study may later be extended to other municipalities in order to be able to shed further light and make inferences at a national scale. The aim was not to have an in-depth analysis of CDPs, but rather to acquire some information on nutrition integration with key informants. Furthermore, the study could not directly include the managers or resource persons in the focus groups from Associations or Non-Governmental Organizations involved in the implementation of nutrition interventions at the local level. However, the key informants who did partake in the study work in direct partnership with them and are familiar with the realities on the ground.

## Conclusions

These results highlight a need reinforce the authority and capacity of mayors and other subnational actors to better incorporate nutrition planning into local development schemes as part of greater measures to support the operationalization of the National Multi-Sector Nutrition Plan. This is critical for enabling and strengthening related multisectoral action undertaken in the municipalities and at village level to holistically address malnutrition and place people at the centre. After this study was concluded, a guide was developed and disseminated through the REACH initiative to facilitate the integration of nutrition into local development plans and strategies. This has been used by national and international institutions in some municipalities for their respective intervention areas as well as for the training of local actors. It has also catalysed the review of CDPs in most municipalities. An assessment of the capacity situation at the mid-term of the strategic plan 2020–2024 is useful to detect subsequent changes and to initiate actions that further improve the organizational capacity dimension, enabling robust nutritional action to be taken at scale.

## Paper context

A number of studies have been carried out on capacity building in nutrition through training curricula and skills development. However, no assessment has yet considered a multisectoral approach focusing on organizational capacity dimensions. Our study is based on a nutrition capacity assessment framework developed by the United Nations Nutrition Network. Our findings represent important insights on municipalities’ organisational capacity for nutrition, and can be useful to improve nutrition impact in the Sahel region.
